# Oral Fucoidan Attenuates Lung Pathology and Clinical Signs in a Severe Influenza A Mouse Model

**DOI:** 10.3390/md18050246

**Published:** 2020-05-08

**Authors:** Claire Richards, Neil A. Williams, J. Helen Fitton, Damien N. Stringer, Samuel S. Karpiniec, Ah Young Park

**Affiliations:** 1 Charles River Laboratories, Bristol BS20 7AW, UK; claire.richards@crl.com; 2 KWS BioTest Ltd., Bristol BS20 7LZ, UK; neil.williams9@icloud.com; 3 Marinova, 249 Kennedy Drive, Cambridge 7170, Australia; damien.stringer@marinova.com.au (D.N.S.); sam.karpiniec@marinova.com.au (S.S.K.); ahyoung.park@marinova.com.au (A.Y.P)

**Keywords:** anti-viral, fucoidan, influenza, inflammation, mice, lung, *Undaria pinnatifida*

## Abstract

Fucoidans are known to be effective inhibitors of inflammation, and of virus binding and cellular entry. *Undaria pinnatifida*-derived fucoidan (UPF) was assessed in a severe influenza A (H1N1, PR8) infection model in mice. Initially, UPF was gavaged at 3.52 mg daily in a treatment model. Gross lung pathology (consolidation) was significantly reduced as compared to controls. UPF was then presented as a feed supplement at a rate of either nil, 3.52 mg/day or 7.04 mg/day in a prophylactic model, dosed three days before infection. A significant improvement was observed in the clinical signs of ill-health, as well as a reduction in gross lung pathology in animals treated with the higher dose, although there was no significant reduction in lung viral titres.

## 1. Introduction

A variety of carbohydrate compounds from marine algae have been shown to exert inhibitory effects on influenza and other respiratory viruses. These compounds include fucoidans [1,2,3,4,5], carrageenans [6,7], ulvans, and some novel carbohydrates from microalgae [8]. These compounds are attractive as dietary supplements to either prevent or reduce the symptoms of influenza and other viral infections. Many are commercially available, are non-toxic, and are components of the normal diet.

The effectiveness of carrageenan polysaccharides against influenza was noted in the 1950s [9], and has recently been extensively refined for activity against respiratory tract viral infections. In that study, the carrageenan was employed intranasally to address the infection at its source. Intranasal carrageenan spray was highly effective against influenza A infection in mice [6,7], and also in double blind clinical trials on the common cold [10]. 

Fucoidans are a class of fucose-rich sulfated carbohydrates found in brown marine algae and echinoderms. They are known to inhibit infection by viruses including Herpes [11,12,13,14,15]. Furthermore, fucoidans have previously been shown to have direct anti-viral activity via the inhibition of viral attachment, entry and replication, against a range of influenza viruses. *Fucus vesiculosus* fucoidan was found to inhibit a wide range of viruses, including parainfluenza type 3 [1]. Fucoidan from *Laminaria japonica* inhibited avian influenza virus infection in pig embryo kidney cultures [16]. Similarly, fucoidan from *Cladosiphon okamuranus* inhibited human para-influenza virus type 2 (hPIV-2) infection in LLCMK(2) cells [3].

In vivo, Hayashi et al. [4] found that inhibition of H1N1 infection in mice could be achieved orally, using a fraction also shown to be an effective inhibitor of other coated viruses. Synytsa et al. [5] found that orally delivered fucoidan was a highly effective inhibitor of avian influenza viruses (H5N3 and H7N2 subtypes) in BALB/c mice. Wang et al. [16] found that a fucoidan from *Kjellmaniella crassifolia* bound toand inhibited the activity of neuraminidase and blocked the release of viral particles. Fucoidan interfered with the activation of EGFR, PKCalpha, NF-kappaB, and Akt, suggesting that it inhibits the cellular EGFR pathway. Intranasal administration of fucoidan improved survival and decreased viral titres in a mouse model. 

*Undaria pinnatifida* fucoidan is a commercially available dietary supplement, and a component of the edible seaweed ‘wakame’. In research by Hayashi [4] and Synytsa [5], an orally delivered—rather than a nasally delivered—high purity fucoidan fraction was an effective treatment for influenza A infection in mice. The fraction used was a well characterised 9 kDa O-acetylated fucogalactan. The effects in the models tested were striking, showing strong stimulation of immunity in addition to a reduction of viral loads. The inhibitory effects were attributable not only to direct inhibition of the virus, but also to the immune response mounted against the virus. Orally delivered fucoidan has been shown to enhance immunity in clinical and animal models. For example, Negishi et al. demonstrated that 300 mg daily of *Undaria pinnatifida* fucoidan, delivered orally, was an effective way to increase the response to seasonal influenza vaccines in elderly subjects [17]. 

In this research we sought to explore whether an orally delivered ‘whole’ fucoidan extract, derived from *Undaria pinnatifida* and exhibiting a broad MW range, was effective in either the treatment or prevention of an influenza infection in a mouse model. The doses chosen (3.52 mg and 7.04 mg) were equivalent to a human dose rate of either ~1 or 2 g daily [18]. 

## 2. Results

### 2.1. Treatment Model: Bodyweight, Clinical Disease Symptoms, and Lung Consolidation Scores

**Figure 1:**
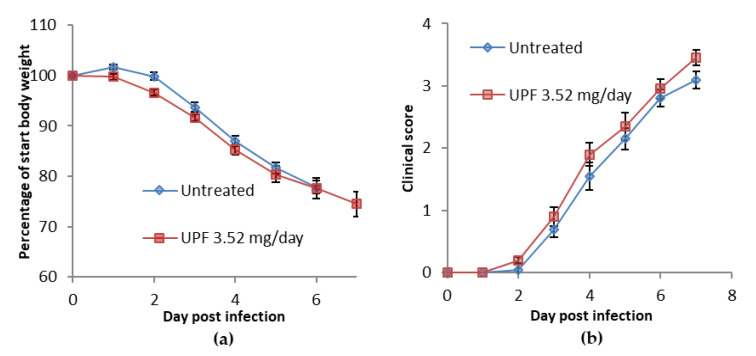
(**a**) Percentage bodyweight change following infection with influenza virus compared with start weight of treated and untreated mice with Undaria pinnatifida fucoidan (UPF). (**b**) Clinical disease scores of treated and untreated mice with UPF following infection with influenza virus. Data are presented as mean per group (n = 10) ± SEM.

During the course of the treatment, in which dosing commenced at the same time as infection, bodyweights continued to decline over the course of the infection. Those animals treated with *Undaria pinnatifida*-derived fucoidan (UPF) showed a slight weight loss, below start weight, from day 2 post-infection which then continued at a similar rate to that observed in the untreated animals (Figure 1a). Clinical disease symptoms were observed in untreated animals from day 3 post-infection. Clinical disease severity increased over the course of the infection in all animals. A similar disease profile was observed in UPF treated animals (Figure 1b).

**Figure 2:**
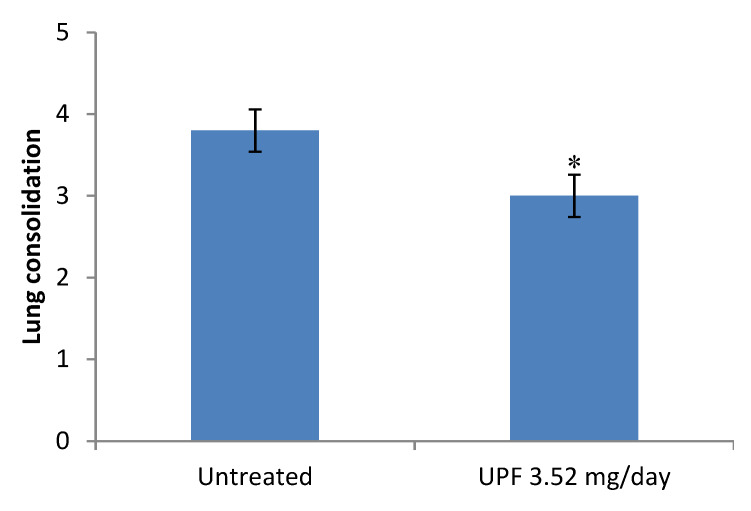
Gross lung pathology at termination following infection with influenza virus. Data are presented as mean per group (n = 10) ± SEM (* *p* < 0.05, unpaired t-test).

**Figure 3:**
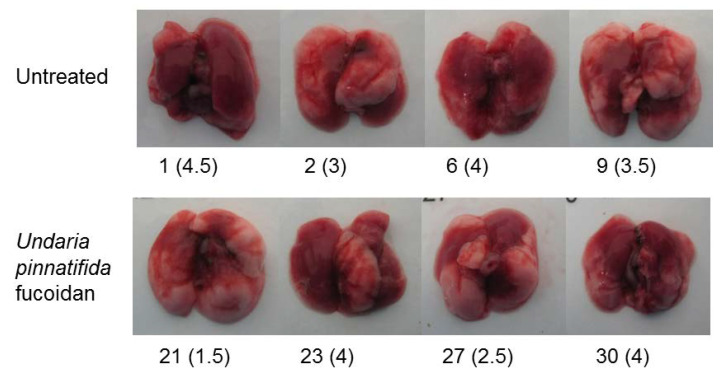
Representative images of lungs at termination following infection with influenza. Ten mice were allotted to either untreated or UPF treated groups. The numbers refer to four random animals in each study group, followed by the score for gross lung pathology, which relates to the area of darker colour.

A slight but significant reduction in gross lung pathology “lung consolidation’’ scores, was observed following termination of animals receiving UPF treatment compared with untreated control animals (Figure 2). Figure 3 shows representative images of lungs at termination after severe influenza virus infection with or without treatment of UPF. Representative histology is shown in Figure S1.

### 2.2. Prevention Model: Bodyweight

**Figure 4:**
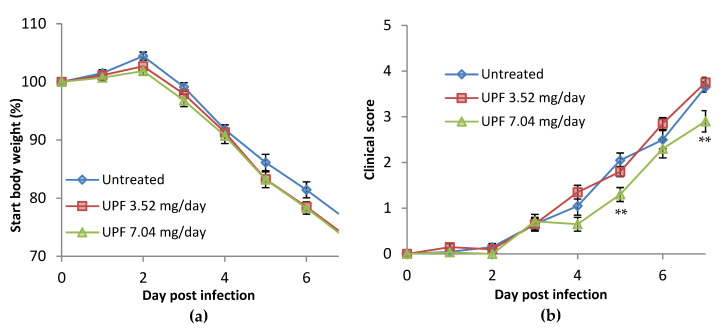
(**a**) Percentage bodyweight change following infection with influenza virus compared with start weight. (**b**) Clinical disease scores following infection with influenza virus. Data are presented as mean per group (n = 10) ± SEM (** *p* < 0.01, unpaired multiple t-test compared with untreated).

In the prevention model, UPF was provided prophylactically in the feed supplement three days prior to infection. Following infection with H1N1 (PR8) Influenza A, untreated mice maintained bodyweight up to day 2 post-infection. From day 3, bodyweight loss was observed, as expected for this model, and continued to decline over the course of the infection. Those animals treated with 3.52 mg/day and 7.04 mg/day of UPF showed a similar weight loss, compared to start weight, from day 3 following infection, which continued at a similar rate to that observed in the untreated animals (Figure 4a).

### 2.3. Prevention Model: Clinical Observations

Clinical disease symptoms were observed in untreated animals from day 3 post-infection. Clinical disease severity increased over the course of the infection in all animals. A similar disease profile was observed in UPF treated animals receiving the lower dose treatment. A significant reduction was observed at the higher dose of 7.04 mg/day at five and seven days post-infection (*p* = 0.0030 and 0.0091, respectively, according to multiple t-test analysis, Figure 4b).

### 2.4. Prevention Model: Lung Consolidation

**Figure 5:**
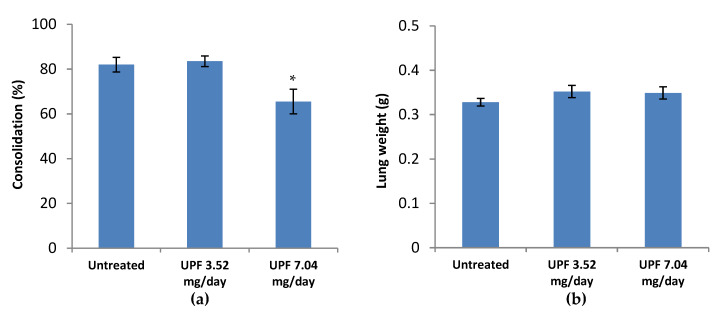
(**a**) Gross lung pathology (consolidation) at termination following infection with influenza virus. Data are presented as mean per group (n = 10) ± SEM. (* *p* < 0.05, unpaired t-test). (**b**) Lung weight at termination following infection with influenza virus. Data are presented as mean per group (n = 10) ± SEM.

A significant reduction in lung consolidation scores was observed following termination of animals receiving the higher dose of UPF (7.04 mg/day) treatment compared with the lower dose (3.52 mg/day) and untreated control animals (*p* = 0.0189), as in Figure 5a. Lung weights were, however, similar for all three groups (Figure 5b).

### 2.5. Prevention Model: Lung Titres

Lung homogenates from individual animals were titrated in triplicate by whole log dilutions down a 96 well plate seeded with MDCK cells, starting at a 10^−1^ dilution. TCID_50_ values were calculated for each sample and group means calculated with SEM (n = 5) A slight reduction in the mean TCID_50_ value of the high-dose UPF treated animals was observed, but this was not a significant reduction (Figure S2, *p* = 0.6942, unpaired t-test).

### 2.6. MTT Viability in the Treatment Model

**Figure 6:**
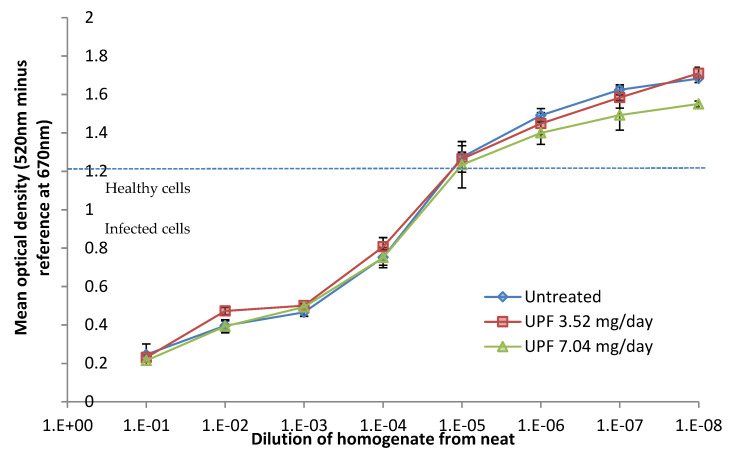
**Madin-Darby canine kidney** (MDCK) cell viability after incubation with serial dilutions of influenza infected lung homogenates. Data are presented as mean optical density (n = 10) ± SEM.

Further analysis by MTT staining showed a slight but non-significant reduction in cell viability in lungs from UPF treated animals (Figure 6).

## 3. Discussion

In the prevention model, the higher oral dose of *Undaria pinnatifida* fucoidan provided a significant level of protection against the clinical signs of H1N1 (PR8) influenza A and lung consolidation in a mouse model when delivered three days prior to infection. In these experiments, there was a lower, but not significant, reduction in viral titres and no significant change in cell viability between the test and control groups of mice. Influenza viruses often replicate not only in lung mucosal tissues, but also in the gut [19]. This activity was confirmed for avian influenza H5N1, for example [20]. Thus oral dosing of fucoidan could address infection directly, via contact with the gut epithelium. It is possible that a higher dose level would significantly reduce viral titres or loads, and this should be assessed in future studies.

The initial treatment study (no pre-treatment with fucoidan) indicated a significant reduction in lung consolidation at 3.52 mg/day delivered by gavage. On histological examination, there was a reduction in perivascular/peribronchiolar lymphoid aggregate formation in the fucoidan-treated mice, although overall histological scores were not significantly different (Supplementary Table S3b, Figure S1). It is also worth noting that the PR8 model is a model of influenza-induced deep lung pneumonia, as opposed to a mild seasonal flu model. We hypothesise that, if the role of fucoidan is primarily in controlling the pathology, it would be particularly applicable to settings where there is virus-associated lung immune pathology. 

Fucoidan is a known selectin blockade agent [21], and may have affected the lymphoid aggregation via this mechanism. Fucoidan uptake and tissue distribution after oral ingestion have been recently demonstrated in a rat model showing preferential accumulation in kidneys and a mean residence time of 6.79 h in serum [22]. Additionally, fucoidan uptake and urinary excretion have been demonstrated in Japanese volunteers [23]. Thus, a small amount of uptake could be assumed, although it remains unclear whether serum-mediated effects are the mechanism of action. The relative contributions of direct anti-viral activity and anti-inflammatory activity or immune modulation are difficult to discern from these data. The changes noted are those where the immunopathology is a key component. The reduced pathology in the absence of an effect on viral titres implies that control of immune pathology was important, and that inhibition of viral replication may be irrelevant. Future studies should investigate higher dosages, measure immunoglobulins, and consider a longer dose of pre-infection dosing.

The *Undaria pinnatifida* fucoidan (UPF) used in this study was prepared by water extraction, without the use of organic solvents, and considered ‘generally recognised as safe’ (GRAS) as a food ingredient and approved in the EU (FDA-notified GRAS: https://www.accessdata.fda.gov/scripts/fdcc/?set=GRASNotices&id=661; https://eur-lex.europa.eu/legal-content/EN/TXT/PDF/?uri=CELEX:32017R2470&from=EN).

It has a broad molecular weight range, and contains a small proportion of alginate and polyphenols as impurities. Analysis relative to dextran standards confirms it is polydisperse with a peak molecular weight of 72.1 kDa. The majority of the sample distribution is found in the 20–1100 kDa range. The extract had a fucoidan purity of 92.8% as determined by various assays described in the experimental section, and contained fucose and galactose in a molar ratio of 1.2:1.0, with a sulfation ratio of 0.74 moles of sulfate per mole of carbohydrate monomer. In addition to the neutral carbohydrates and sulfate, the fucoidan’s charge was balanced by 7.0% w/w cations (comprising K, Na, Ca and Mg) and was naturally acetylated at 2.2% w/w.

By comparison, the experiments that were carried out by Synytsa et al. [5] also used an extract of *Undaria pinnatifida* fucoidan—initially described by Lee et al. [14]—which was extensively purified through a series of ethanol precipitations and column purifications to produce a specific sub-fraction. This fraction was composed of fucose and galactose with an approximate ratio of 1.0:1.1. The degree of substitution of sulfate was 0.72 and its apparent molecular weight was 9 kDa. Synytsa et al. used spectroscopic methods (FTIR, FT Raman and NMR) and defined the fraction as ‘O-acetylated sulfated fucogalactan’, a highly branched complex (1→3)(1→4)- α-fuco-(1→3)(1→4)(1→6)- β-galactan. 

Fucoidans are highly complex, branched molecules. The highly effective fraction used by Synytsa et al. is a unique sub fraction derived from a hydrolysis and purification of the whole fraction. In Synyta et al.’s experiment, the fucoidan fraction was given by oral administration at a dose of 1 mg or 5 mg/day, twice a day, for 14 days: from seven days before virus inoculation until seven days after inoculation. Virus production in BALF, and antibody levels in sera, BALFs and faeces, were all substantively changed, in a dose dependent manner, by the oral dosing of the fraction.

The experiments described in this paper used a similar scale of dosing: 3.52 mg/day or 7.04 mg/day, beginning three days prior to infection and continuing through the infection period. Although viral titres were not significantly altered, clinical signs and lung consolidation were significantly reduced at the higher dose level.

## 4. Experimental Section

### 4.1. Compounds and Reagents—Fucoidan from Undaria Pinnatifida

Fucoidan extract from *Undaria pinnatifida* was provided by Marinova Pty Ltd. The proprietary aqueous extract was designed for ingestion, with a standardised fucoidan content greater than 85% as determined by previously reported methods [24]. The carbohydrate profile was determined using a GC-based method for the accurate determination of individual monosaccharide ratios in a sample. This method relies on the preparation of acetylated alditol derivatives of the hydrolysed samples [25]. The uronic acid content was determined by spectrophotometric analysis of the hydrolysed compound in the presence of 3-phenylphenol, based on a method described by Filisetti-Cozzi and Carpita [26]. Sulfate content was analysed spectrophotometrically using a BaSO_4_ precipitation method (BaCl_2_ in gelatin), based on the work of Dodgson [27,28]. Cations, including Na, K, Ca, and Mg, were determined by Flame Atomic Absorption Spectroscopy.

### 4.2. Delivery of Fucoidan

In-life experimental procedures conducted during the course of the study were evaluated and approved by the University of Bristol Ethics Review Process (ERP) in accordance with the United Kingdom Animals (Scientific Procedures) Act 1986 (2013 amendment), under the Project licence 30/3164 (May 2014). This act, administered by the UK Home Office, regulates all scientific procedures in living animals and conforms to the European Convention for the Protection of Vertebrate Animals Used for Experimental and Other Scientific Purposes (Strasbourg, Council of Europe). 

For the treatment model, *Undaria pinnatifida* fucoidan was reconstituted at 17.6 mg/mL in distilled water. The solution was heated in a water bath to 25 °C for 20 min with agitation. Animals received 0.1 mL twice daily by gavage, to give a daily dose of 3.52 mg per day. 

For the prevention experiment, individual portions of 3 g of wet mass of food were prepared (in powdered form, which can be ground up pellets of food bought as powder). The food contained a defined amount of *Undaria pinnatifida* fucoidan per day (mg/kg for average mouse weight, either 3.52 mg/day or 7.04 mg/day) and some sucrose (4% w/w). The food was frozen on small plastic balance trays. Animals were trained in food administration by starting with food trays that did not contain the drug. Due to the sucrose content of the food portion, they preferentially ate this food first. Normal food pellets were also available. A frozen tray of food from the freezer was taken each day and dropped into the cage at the same time in the evening. At the same time, empty plastic trays from the day before were removed from the cage. 

Influenza A/Puerto Rico/8/1934 (H1N1, PR8) was applied intranasally under gaseous anaesthesia of isoflurane and oxygen. The virus was diluted 1 in 100,000 in PBS and administered in a volume of 35 μL pipetted on to the tip of the nose and inhaled. Animals were placed on their backs in the cage to allow infection and to recover.

### 4.3. Assessment of Fucoidan from Undaria pinnatifida in a Treatment Model

Female BALB/c mice were allocated to experimental groups and allowed to acclimatise for one week. On day 0, animals were given a single intra-nasal dose of H1N1 influenza virus under isoflurane anaesthesia. Immediately following infection, animals were treated by oral gavage. All animals were terminated seven days post-infection. Signs of clinical disease and weight loss were scored daily. At termination, lungs were inspected for pathology and weighed. 

### 4.4. Assessment of Fucoidan from Undaria Pinnatifida in a Prevention Model

Female BALB/c mice were allocated to experimental groups and allowed to acclimatise for a minimum of one week. Ten mice each were allotted to each treatment group, with an additional five animals per treatment group allotted to ‘satellite groups’ to be terminated at day 3. Animals were transferred into individual cages and fed prepared food trays as described above, without the drug. From three days prior to infection, mice were treated with prepared food trays with or without fucoidan. On day 0, all animals were given a single intra-nasal dose of H1N1 PR8 influenza virus under isoflurane anaesthesia. On day 3 post-infection, animals from satellite groups for each treatment condition were terminated and their lungs were assessed for viral titres by TCID_50_ assay on MDCK cells followed by an MTT assay. Signs of clinical disease and weight loss were scored daily and times at which humane endpoints were reached, leading to termination, were recorded. On day 7 the surviving animals were terminated and lungs were inspected for pathology and weighed.

### 4.5. Assessment of Clinical Symptoms

All animals were monitored daily for signs of infection, including reduced activity, abnormal posture, piloerection, ocular discharge, and laboured breathing. Any other abnormalities were also recorded. Each of these parameters were scored semi-quantitatively according to a routine clinical scoring system. Bodyweight was recorded prior to infection and daily thereafter to enable the percentage change in bodyweight to be determined. Data were provided for all live animals throughout the time-course.

### 4.6. Lung Consolidation and Histopathology

For both treatment and prevention models, the lungs were assessed at termination for signs of consolidation. The lungs of infected mice show signs of viral damage leading to haemorrhage, necrosis and immune cell infiltration. Together these changes resulted in areas of the lung acquiring a plum colouration which were photographed and scored on a semi-quantitative scale (0–5), represented in Figure 2 and Figure 5a. Representative images are provided in Figure 3. 

Lungs were placed in tissue fixative at termination. Two standard sections were taken from two contralateral lung lobes in standard fashion. These were processed routinely and embedded in paraffin wax. Sections were stained with haematoxylin and eosin and scored in a blinded fashion. The most severely affected portion of tissue on each slide was scored. The scoring system was adapted from Schouten et al. [29] and Longhi et al. [30]. The following parameters were each scored on a four point scale (where 0 = normal, 1 = mild, 2 = moderate, and 3 = severe): bronchitis or bronchiolitis (including epithelial necrosis); interstitial inflammation; alveolitis; pleuritis; haemorrhage; perivascular/peribronchiolar lymphoid aggregate formation. The maximum possible histopathology score was 3. Additionally, the approximate percentage area of tissue affected in the most severely affected section of lung for each animal was determined. See Supplementary Tables for details.

## 5. Conclusions

Orally delivered fucoidan from *Undaria pinnatifida* significantly reduced gross lung pathology (consolidation) in a BALB/c mouse model of severe H1N1 (PR8) influenza, when administered at the same time as the viral infection. When the fucoidan was included in a feed supplement three days prior to infection, both clinical signs of influenza and gross lung pathology were reduced in a dose-dependent manner. In order to significantly reduce clinical scores and lung consolidation, the higher dose—equivalent to a human dose of about 2 g daily—was required. Although viral titres were lowered, these were not significant. Inhibition of inflammation and immune regulation, although not measured directly here, may account for the observed effects. *Undaria pinnatifida* fucoidan is a known entry inhibitor of the H1N1 influenza virus [14]. Though not directly measured, inhibition of viral load in the gut rather than lung mucosa may have resulted in the reduction in clinical symptoms. 

The reduction in symptoms and lung consolidation in this model indicates the potential utility of this type of edible *Undaria pinnatifida* fucoidan as a nutritional supplement in the management of acute viral respiratory infection. Future work should investigate the potential for further reductions in symptoms using higher doses of fucoidan.

## Supplementary Materials

The following are available online at https://www.mdpi.com/1660-3397/18/5/246/s1, Table S1: Bodyweight in treatment model, Table S2: Clinical observation in treatment model, Table S3a: Lung pathology in treatment model, Table S3b: Lung histopathology in treatment model, Table S4: Bodyweight in prevention model, Table S5: Clinical observations in prevention model, Table S6: Lung pathology in prevention model, Table S7: TCID50 scores in prevention model, Table S8: MTT optical density in prevention model, Figure S1:Representative images of lung histopathology scores and area affected at termination in treatment model, Figure S2: Mean TCID_50_ values of lung homogenates. Data are presented as mean TCID_50_ of group (n = 5) ± SEM.
