# Supplementary material for: Oral Fucoidan Attenuates Lung Pathology and Clinical Signs in a Severe Influenza A Mouse Model
Source: Mar Drugs. 2020 May 8;18(5):246. doi: 10.3390/md18050246 (PMC7281568; doi:10.3390/md18050246)

Supplementary Information

Fucoidan decreases the severity of influenza A infection in a mouse model

Claire Richards [1], Neil A. Williams [2], Janet Helen Fitton [3,*], Damien N. Stringer[3], Samuel S. Karpiniec[3], Ah Young Park[3]

[1] Charles River Laboratories, Bristol, BS20 7AW, UK; claire.richards@crl.com
[2] KWS BioTest Ltd., Bristol, BS20 7AW, UK; neil.williams9@icloud.com
[3] Marinova, 249 Kennedy Drive, Cambridge, 7170Australia; damien.stringer@marinova.com.au (D.N.S.); sam.karpiniec@marinova.com.au (S.S.K.), ahyoung.park@marinova.com.au (A.Y.P)
* Correspondence: helen.fitton@marinova.com.au; Tel.: +61-3-6248-5800; Fax: +61-3-6248-4062.

Contents

Table S1	Bodyweight in treatment model	Page 3
Table S2	Clinical observation in treatment model	Page 3
Table S3a	Lung pathology in treatment model	Page 4
Table S3b	Lung histopathology in treatment model	Page 4
Table S4	Bodyweight in prevention model	Page 5
Table S5	Clinical observations in prevention model	Page 6
Table S6	Lung pathology in prevention model	Page 7
Table S7	$TCID_{50}$ scores in prevention model	Page 8
Table S8	MTT optical density in prevention model	Page 11
Figure S1.	Representative images of lung histopathology scores and area affected at termination in treatment model.	Page 17
Figure S2.	Mean $TCID_{50}$ values of lung homogenates. Data are presented as mean $TCID_{50}$ of group (n = 5) ± SEM.	Page 17

Table S1.Bodyweight in treatment model.

Bodyweights (g) (%)		weight (g)								% start weight							
		0	1	2	3	4	5	6	7	0	1	2	3	4	5	6	7
Untreated	1	19	18.8	18.3	16.9	15.5	14.4	13.5	12.8	100	98.9	96.3	88.9	81.6	75.8	71.1	67.4
Untreated	2	19.3	19.6	19.4	18.4	17.2	15.6	14.8	14.1	100	101.6	100.5	95.3	89.1	80.8	76.7	73.1
Untreated	3	19.6	20.3	19.9	18.6	17.4	16.5	15.7	14.8	100	103.6	101.5	94.9	88.8	84.2	80.1	75.5
Untreated	4	18.2	18.1	17.5	16.1	14.9	13.8	13.1	12.3	100	99.5	96.2	88.5	81.9	75.8	72.0	67.6
Untreated	5	19.9	20.3	20.1	19.7	18.3	17	16.6	15.3	100	102.0	101.0	99.0	92.0	85.4	83.4	76.9
Untreated	6	19.6	19.7	19.7	18.7	16.8	16	15.3	14.3	100	100.5	100.5	95.4	85.7	81.6	78.1	73.0
Untreated	7	19.2	19.6	19.2	17.6	16.8	16	15	14.2	100	102.1	100.0	91.7	87.5	83.3	78.1	74.0
Untreated	8	18.4	19.1	18.6	17.3	16.3	15.9	15.2	14.4	100	103.8	101.1	94.0	88.6	86.4	82.6	78.3
Untreated	9	17.9	18.3	17.5	16.8	15.8	14.6	14	13.3	100	102.2	97.8	93.9	88.3	81.6	78.2	74.3
Untreated	10	16.3	16.8	16.9	15.6	14.2	13.3	12.7	12	100	103.1	103.7	95.7	87.1	81.6	77.9	73.6
UPF 3.52 mg/day	21	18.5	18.9	18.2	16.7	15.7	14.7	14.2	13.5	100	102.2	98.4	90.3	84.9	79.5	76.8	73.0
UPF 3.52 mg/day	22	20.4	20.6	19.9	18.5	16.8	15.4	14.5	13.8	100	101.0	97.5	90.7	82.4	75.5	71.1	67.6
UPF 3.52 mg/day	23	19	19	18.6	17.5	15.8	14.9	14.1	13.4	100	100.0	97.9	92.1	83.2	78.4	74.2	70.5
UPF 3.52 mg/day	24	17.5	17.2	16.6	15.7	14.6	13.7	13	12.4	100	98.3	96.5	94.6	93.0	93.8	94.9	95.4
UPF 3.52 mg/day	25	16.9	16.9	16.3	15.1	13.8	13	12.5	11.7	100	100.0	96.4	89.3	81.7	76.9	74.0	69.2
UPF 3.52 mg/day	26	18.7	19.2	18.5	17.9	16.4	15.3	14.3	14	100	102.7	98.9	95.7	87.7	81.8	76.5	74.9
UPF 3.52 mg/day	27	17.7	17.1	16.9	16.1	14.9	13.9	13.5	12.9	100	96.6	95.5	91.0	84.2	78.5	76.3	72.9
UPF 3.52 mg/day	28	20	19.9	19.5	18.4	17.4	16.3	16	15.1	100	99.5	97.5	92.0	87.0	81.5	80.0	75.5
UPF 3.52 mg/day	29	20.5	20.1	19.1	18.7	17.3	16.2	15.6	14.9	100	98.0	93.2	91.2	84.4	79.0	76.1	72.7
UPF 3.52 mg/day	30	18.3	18.1	17.2	16.4	15.4	14.4	14	13.4	100	98.9	94.0	89.6	84.2	78.7	76.5	73.2

Red cells bodyweights <90%, yellow cells bodyweight <80%.

Table S2. Clinical observations in treatment model.

Day		0	1	2	3	4	5	6	7
Untreated	1	0	0	0.5	0.5	2.5	3	3	3
Untreated	2	0	0	0	0	0.5	1	3	3
Untreated	3	0	0	0	0.5	2	3	3	3.5
Untreated	4	0	0	0	1.5	2	3	3.5	3.5
Untreated	5	0	0	0	0.5	1.5	2	2	3.5
Untreated	6	0	0	0	0.5	1	2	3	2.5
Untreated	7	0	0	0	0.5	1.5	1.5	2	2.5
Untreated	8	0	0	0	0.5	2	2	2.5	3.5
Untreated	9	0	0	0	1	1	2.5	3	3
Untreated	10	0	0	0	1.5	1.5	1.5	3	3
UPF 3.52 mg/day	21	0	0	0	0.5	2.5	2	2.5	3
UPF 3.52 mg/day	22	0	0	0	1	2.5	3	3	4
UPF 3.52 mg/day	23	0	0	0.5	1	1	3	3	3.5
UPF 3.52 mg/day	24	0	0	0.5	1	3	2.5	3.5	4
UPF 3.52 mg/day	25	0	0	0	1	2	3	2.5	4
UPF 3.52 mg/day	26	0	0	0	1.5	1.5	2	3.5	3.5
UPF 3.52 mg/day	27	0	0	0.5	0.5	1.5	2.5	2.5	3
UPF 3.52 mg/day	28	0	0	0	0.5	1.5	2	3.5	3
UPF 3.52 mg/day	29	0	0	0	1.5	1	2	3	3
UPF 3.52 mg/day	30	0	0	0.5	0.5	2.5	1.5	2.5	3.5

Scoring system 0-5 based on incidence of piloerection, hunched posture, abnormal breathing (to include rapid, irregular or slowed breathing), impaired mobility and ocular discharge. Each parameter is given a score of 0 = not present, 0.5 = partially and 1 = present. The scores are added together to give a maximum score of 5.

Table S3a. Lung pathology in treatment model.

		% lung pathology	Lung path (0-5)	Lung weight
**Untreated**	**1**	90	4.5	0.36
**Untreated**	**2**	60	3	0.316
**Untreated**	**3**	80	4	0.336
**Untreated**	**4**	80	4	0.354
**Untreated**	**5**	80	4	0.346
**Untreated**	**6**	90	4.5	0.328
**Untreated**	**7**	60	3	0.294
**Untreated**	**8**	60	3	0.278
**Untreated**	**9**	70	3.5	0.278
**Untreated**	**10**	90	4.5	0.288
**UPF 3.52 mg/day**	**21**	30	1.5	0.384
**UPF 3.52 mg/day**	**22**	60	3	0.408
**UPF 3.52 mg/day**	**23**	80	4	0.256
**UPF 3.52 mg/day**	**24**	60	3	0.346
**UPF 3.52 mg/day**	**25**	70	3.5	0.298
**UPF 3.52 mg/day**	**26**	40	2	0.316
**UPF 3.52 mg/day**	**27**	50	2.5	0.33
**UPF 3.52 mg/day**	**28**	60	3	0.308
**UPF 3.52 mg/day**	**29**	70	3.5	0.308
**UPF 3.52 mg/day**	**30**	80	4	0.31

Table S3b. Lung histopathology in treatment model.

Slide ID number [animal number]	Bronchitis	Interstit	Alveolitis	Pleuritis	Haemorrhage	PV Lymphoid	Total Pathology	% Area
E0161-14 [1.1]	2	1	1	0	0	0	4	50
E0162-14 [1.2]	2	2	1	0	1	1	7	75
E0163-14 [1.3]	3	2	1	0	3	0	9	75
E0164-14 [1.4]	1	2	1	1	0	1	6	50
E0165-14 [1.5]	1	2	1	0	0	1	5	50
E0166-14 [1.6]	2	2	1	0	0	1	6	50
E0167-14 [1.7]	2	2	1	1	1	1	8	80
E0168-14 [1.8]	2	3	1	0	1	1	8	100
E0169-14 [1.9]	2	2	1	1	1	1	8	50
E0170-14 [1.10]	2	2	1	0	0	0	5	75
E0181-14 [3.21]	3	2	1	0	1	1	8	50
E0182-14 [3.22]	2	2	1	0	0	0	5	90
E0183-14 [3.23]	2	2	1	0	0	0	5	90
E0184-14 [3.24]	2	3	1	1	0	0	7	50
E0185-14 [3.25]	2	2	1	0	1	0	6	80
E0186-14 [3.26]	2	2	1	0	0	1	6	75
E0187-14 [3.27]	2	2	1	0	0	0	5	50
E0188-14 [3.28]	2	2	1	1	0	0	6	75
E0189-14 [3.29]	2	2	1	0	1	0	6	90
E0190-14 [3.30]	2	2	1	0	0	0	5	60

Table S4. Bodyweight in prevention model

		0	1	2	3	4	5	6	7	0	1	2	3	4	5	6	7
1. Untreated	1.1	20.1	20.1	20.6	20.3	19.1	19.1	18.1	16.5	100.0	100.0	102.5	101.0	95.0	95.0	90.0	82.1
1. Untreated	1.2	17.1	17.6	18.1	16.9	15.2	13.6	13.0	12.4	100.0	102.9	105.8	98.8	88.9	79.5	76.0	72.5
1. Untreated	1.3	17.8	17.9	18.0	17.4	15.9	14.8	14.0	13.5	100.0	100.6	101.1	97.8	89.3	83.1	78.7	75.8
1. Untreated	1.4	17.1	17.5	18.1	17.5	16.4	14.9	14.1	13.2	100.0	102.3	105.8	102.3	95.9	87.1	82.5	77.2
1. Untreated	1.5	16.8	17.0	17.2	16.5	15.4	15.0	14.3	13.2	100.0	101.2	102.4	98.2	91.7	89.3	85.1	78.6
1. Untreated	1.6	16.4	17.0	17.7	16.6	15.3	14.5	13.4	12.5	100.0	103.7	107.9	101.2	93.3	88.4	81.7	76.2
1. Untreated	1.7	17.8	17.9	18.3	16.9	15.8	15.0	13.8	13.3	100.0	100.6	102.8	94.9	88.8	84.3	77.5	74.7
1. Untreated	1.8	16.3	17.1	17.4	16.0	14.6	13.2	12.5	12.0	100.0	104.9	106.7	98.2	89.6	81.0	76.7	73.6
1. Untreated	1.9	19.1	19.1	20.1	19.4	17.8	16.7	15.8	14.4	100.0	100.0	105.2	101.6	93.2	87.4	82.7	75.4
1. Untreated	1.10	18.0	17.8	18.7	17.5	16.6	15.5	15.0	13.7	100.0	98.9	103.9	97.2	92.2	86.1	83.3	76.1
1. Untreated satellite	1.11	17.0	16.7	17.6	16.3					100.0	98.2	103.5	95.9				
1. Untreated satellite	1.12	17.8	17.7	18.0	16.7					100.0	99.4	101.1	93.8				
1. Untreated satellite	1.13	19.7	19.6	19.9	19.1					100.0	99.5	101.0	97.0				
1. Untreated satellite	1.14	17.5	17.8	18.4	16.6					100.0	101.7	105.1	94.9				
1. Untreated satellite	1.15	17.3	17.6	17.7	17.0					100.0	101.7	102.3	98.3				
2. Undaria Fucoidan 3.52mg/day	2.16	18.2	18.2	18.7	18.3	16.8	15.6	14.5	13.6	100.0	100.0	102.7	100.5	92.3	85.7	79.7	74.7
2. Undaria Fucoidan 3.52mg/day	2.17	18.8	18.5	18.8	18.3	17.1	15.8	15.3	14.2	100.0	98.4	100.0	97.3	91.0	84.0	81.4	75.5
2. Undaria Fucoidan 3.52mg/day	2.18	20.3	20.7	20.7	20.0	18.2	16.2	15.4	14.4	100.0	102.0	102.0	98.5	89.7	79.8	75.9	70.9
2. Undaria Fucoidan 3.52mg/day	2.19	17.4	17.8	18.7	17.5	16.5	14.8	13.7	12.8	100.0	102.3	107.5	100.6	94.8	85.1	78.7	73.6
2. Undaria Fucoidan 3.52mg/day	2.20	19.1	19.1	19.5	18.4	17.4	15.9	15.4	14.1	100.0	100.0	102.1	96.3	91.1	83.2	80.6	73.8
2. Undaria Fucoidan 3.52mg/day	2.21	18.8	19.0	19.0	17.8	16.5	15.0	14.0	12.8	100.0	101.1	101.1	94.7	87.8	79.8	74.5	68.1
2. Undaria Fucoidan 3.52mg/day	2.22	19.9	20.0	20.2	19.4	18.5	17.3	16.0	14.7	100.0	100.5	101.5	97.5	93.0	86.9	80.4	73.9
2. Undaria Fucoidan 3.52mg/day	2.23	16.7	17.1	17.0	16.4	15.4	14.0	13.4	12.8	100.0	102.4	101.8	98.2	92.2	83.8	80.2	76.6
2. Undaria Fucoidan 3.52mg/day	2.24	16.2	16.8	17.1	15.8	14.6	13.1	12.2	11.8	100.0	103.7	105.6	97.5	90.1	80.9	75.3	72.8
2. Undaria Fucoidan 3.52mg/day	2.25	19.6	19.4	20.5	19.1	17.6	15.8	14.8	13.8	100.0	99.0	104.6	97.4	89.8	80.6	75.5	70.4
2. Undaria Fucoidan 3.52mg/day satellite	2.26	17.9	18.4	18.6	17.9					100.0	102.8	103.9	100.0				
2. Undaria Fucoidan 3.52mg/day satellite	2.27	19.4	19.4	19.4	18.3					100.0	100.0	100.0	94.3				
2. Undaria Fucoidan 3.52mg/day satellite	2.28	18.3	18.5	18.6	18.1					100.0	101.1	101.6	98.9				
2. Undaria Fucoidan 3.52mg/day satellite	2.29	17.8	17.8	18.0	16.7					100.0	100.0	101.1	93.8				
2. Undaria Fucoidan 3.52mg/day satellite	2.30	18.1	17.7	18.3	17.1					100.0	97.8	101.1	94.5				
3. Undaria Fucoidan 7.04mg/day	3.31	19.5	19.5	19.3	18.1	16.8	15.5	14.6	13.8	100.0	100.0	99.0	92.8	86.2	79.5	74.9	70.8
3. Undaria Fucoidan 7.04mg/day	3.32	19.7	19.8	20.1	18.8	17.0	15.7	14.8	13.6	100.0	100.5	102.0	95.4	86.3	79.7	75.1	69.0
3. Undaria Fucoidan 7.04mg/day	3.33	19.1	18.8	18.8	17.3	16.1	14.8	14.4	13.7	100.0	98.4	98.4	90.6	84.3	77.5	75.4	71.7
3. Undaria Fucoidan 7.04mg/day	3.34	18.9	19.6	19.6	18.3	17.3	16.1	15.2	14.1	100.0	103.7	103.7	96.8	91.5	85.2	80.4	74.6
3. Undaria Fucoidan 7.04mg/day	3.35	18.8	19.2	19.3	18.4	17.0	15.1	14.3	13.3	100.0	102.1	102.7	97.9	90.4	80.3	76.1	70.7
3. Undaria Fucoidan 7.04mg/day	3.36	18.6	18.5	19.0	17.9	16.8	15.3	14.3	13.5	100.0	99.5	102.2	96.2	90.3	82.3	76.9	72.6
3. Undaria Fucoidan 7.04mg/day	3.37	19.9	20.0	20.8	20.1	19.1	17.7	16.4	15.0	100.0	100.5	104.5	101.0	96.0	88.9	82.4	75.4
3. Undaria Fucoidan 7.04mg/day	3.38	19.7	20.2	20.6	20.0	19.2	17.9	16.7	15.4	100.0	102.5	104.6	101.5	97.5	90.9	84.8	78.2
3. Undaria Fucoidan 7.04mg/day	3.39	18.7	18.9	18.9	18.1	17.2	15.4	14.5	13.4	100.0	101.1	101.1	96.8	92.0	82.4	77.5	71.7
3. Undaria Fucoidan 7.04mg/day	3.40	18.2	18.0	18.3	18.0	16.9	15.5	14.5	13.6	100.0	98.9	100.5	98.9	92.9	85.2	79.7	74.7
3. Undaria Fucoidan 7.04mg/day satellite	3.41	16.9	17.0	17.4	16.5					100.0	100.6	103.0	97.6				
3. Undaria Fucoidan 7.04mg/day satellite	3.42	18.7	19.1	19.8	18.0					100.0	102.1	105.9	96.3				
3. Undaria Fucoidan 7.04mg/day satellite	3.43	18.0	18.2	19.1	18.3					100.0	101.1	106.1	101.7				
3. Undaria Fucoidan 7.04mg/day satellite	3.44	17.7	17.9	18.9	17.9					100.0	101.1	106.8	101.1				
3. Undaria Fucoidan 7.04mg/day satellite	3.45	17.1	17.3	17.6	16.1					100.0	101.2	102.9	94.2				

Table S5. Clinical observations in prevention model

		0	1	2	3	4	5	6	7
1. Untreated	1.1	0.0	0.0	0.0	0.5	0.5	1.0	2.5	3.5
1. Untreated	1.2	0.0	0.0	0.5	1.0	2.0	2.5	3.0	4.0
1. Untreated	1.3	0.0	0.0	0.0	0.5	0.5	2.5	2.5	3.5
1. Untreated	1.4	0.0	0.0	0.0	1.5	1.5	2.5	3.0	4.0
1. Untreated	1.5	0.0	0.0	0.0	0.5	0.5	1.5	1.5	3.5
1. Untreated	1.6	0.0	0.0	0.0	1.0	1.0	2.0	2.0	3.0
1. Untreated	1.7	0.0	0.0	0.5	1.0	1.5	2.0	3.0	3.5
1. Untreated	1.8	0.0	0.0	0.0	0.0	1.5	2.5	3.0	3.5
1. Untreated	1.9	0.0	0.0	0.0	0.0	0.0	2.0	1.5	4.0
1. Untreated	1.10	0.0	0.5	0.5	0.5	1.5	2.0	3.0	4.0
1. Untreated satellite	1.11	0.0	0.0	0.0	1.0				
1. Untreated satellite	1.12	0.0	0.0	0.0	0.5				
1. Untreated satellite	1.13	0.0	0.0	0.0	0.5				
1. Untreated satellite	1.14	0.0	0.0	0.0	1.5				
1. Untreated satellite	1.15	0.0	0.5	0.5	1.0				
2. Undaria Fucoidan 3.52mg/day	2.16	0.0	0.0	0.0	1.0	1.5	2.0	2.5	4.0
2. Undaria Fucoidan 3.52mg/day	2.17	0.0	0.5	0.0	0.5	0.5	1.5	2.5	3.5
2. Undaria Fucoidan 3.52mg/day	2.18	0.0	0.0	0.0	0.5	1.5	2.0	2.5	4.0
2. Undaria Fucoidan 3.52mg/day	2.19	0.0	0.0	0.0	0.0	1.0	2.0	3.0	4.0
2. Undaria Fucoidan 3.52mg/day	2.20	0.0	0.5	0.5	1.0	1.5	2.0	3.0	3.5
2. Undaria Fucoidan 3.52mg/day	2.21	0.0	0.0	0.0	0.5	1.5	2.0	3.5	3.0
2. Undaria Fucoidan 3.52mg/day	2.22	0.0	0.5	0.0	0.0	1.0	1.0	2.5	4.0
2. Undaria Fucoidan 3.52mg/day	2.23	0.0	0.0	0.5	1.0	2.0	2.0	3.0	4.0
2. Undaria Fucoidan 3.52mg/day	2.24	0.0	0.0	0.0	1.0	1.0	2.0	3.5	4.0
2. Undaria Fucoidan 3.52mg/day	2.25	0.0	0.0	0.0	1.0	2.0	1.5	2.5	3.5
2. Undaria Fucoidan 3.52mg/day satellite	2.26	0.0	0.0	0.0	0.5				
2. Undaria Fucoidan 3.52mg/day satellite	2.27	0.0	0.0	0.0	0.5				
2. Undaria Fucoidan 3.52mg/day satellite	2.28	0.0	0.0	0.0	0.5				
2. Undaria Fucoidan 3.52mg/day satellite	2.29	0.0	0.0	0.0	1.5				
2. Undaria Fucoidan 3.52mg/day satellite	2.30	0.0	0.5	0.0	0.5				
3. Undaria Fucoidan 7.04mg/day	3.31	0.0	0.0	0.0	1.0	0.5	2.0	2.5	4.0
3. Undaria Fucoidan 7.04mg/day	3.32	0.0	0.0	0.0	1.5	0.5	1.5	2.5	3.0
3. Undaria Fucoidan 7.04mg/day	3.33	0.0	0.0	0.0	1.0	1.0	1.0	3.5	3.5
3. Undaria Fucoidan 7.04mg/day	3.34	0.0	0.0	0.0	0.5	1.0	1.5	2.5	3.0
3. Undaria Fucoidan 7.04mg/day	3.35	0.0	0.0	0.0	0.0	1.5	2.0	2.0	3.5
3. Undaria Fucoidan 7.04mg/day	3.36	0.0	0.0	0.0	0.5	0.0	0.5	2.0	1.5
3. Undaria Fucoidan 7.04mg/day	3.37	0.0	0.0	0.0	0.0	0.0	1.0	3.0	2.5
3. Undaria Fucoidan 7.04mg/day	3.38	0.0	0.0	0.0	1.0	1.0	1.5	2.0	3.0
3. Undaria Fucoidan 7.04mg/day	3.39	0.0	0.0	0.0	0.5	0.5	1.0	1.5	3.0
3. Undaria Fucoidan 7.04mg/day	3.40	0.0	0.0	0.0	1.0	0.5	1.0	1.5	2.0
3. Undaria Fucoidan 7.04mg/day satellite	3.41	0.0	0.0	0.0	1.0				
3. Undaria Fucoidan 7.04mg/day satellite	3.42	0.0	0.5	0.0	1.5				
3. Undaria Fucoidan 7.04mg/day satellite	3.43	0.0	0.0	0.5	0.5				
3. Undaria Fucoidan 7.04mg/day satellite	3.44	0.0	0.0	0.0	1.0				
3. Undaria Fucoidan 7.04mg/day satellite	3.45	0.0	0.0	0.5	1.5				

Scoring system 0-5 based on incidence of piloerection, hunched posture, abnormal breathing (to include rapid, irregular or slowed breathing), impaired mobility and ocular discharge. Each parameter is given a score of 0 = not present, 0.5 = partially and 1 = present. The scores are added together to give a maximum score of 5.

Table S6. Lung pathology in prevention model.

		Lung pathology	Lung weight
1. Untreated	1.1	85	0.340
1. Untreated	1.2	90	0.335
1. Untreated	1.3	75	0.295
1. Untreated	1.4	75	0.330
1. Untreated	1.5	90	0.290
1. Untreated	1.6	60	0.370
1. Untreated	1.7	80	0.295
1. Untreated	1.8	90	0.325
1. Untreated	1.9	95	0.355
1. Untreated	1.10	80	0.345
2. Undaria Fucoidan 3.52mg/day	2.16	90	0.330
2. Undaria Fucoidan 3.52mg/day	2.17	80	0.315
2. Undaria Fucoidan 3.52mg/day	2.18	75	0.425
2. Undaria Fucoidan 3.52mg/day	2.19	85	0.360
2. Undaria Fucoidan 3.52mg/day	2.20	95	0.400
2. Undaria Fucoidan 3.52mg/day	2.21	90	0.370
2. Undaria Fucoidan 3.52mg/day	2.22	75	0.350
2. Undaria Fucoidan 3.52mg/day	2.23	75	0.270
2. Undaria Fucoidan 3.52mg/day	2.24	90	0.335
2. Undaria Fucoidan 3.52mg/day	2.25	80	0.365
3. Undaria Fucoidan 7.04mg/day	3.31	30	0.380
3. Undaria Fucoidan 7.04mg/day	3.32	80	0.410
3. Undaria Fucoidan 7.04mg/day	3.33	70	0.295
3. Undaria Fucoidan 7.04mg/day	3.34	50	0.320
3. Undaria Fucoidan 7.04mg/day	3.35	60	0.340
3. Undaria Fucoidan 7.04mg/day	3.36	75	0.335
3. Undaria Fucoidan 7.04mg/day	3.37	60	0.335
3. Undaria Fucoidan 7.04mg/day	3.38	80	0.395
3. Undaria Fucoidan 7.04mg/day	3.39	90	0.395
3. Undaria Fucoidan 7.04mg/day	3.40	60	0.285

Table S7. $TCID_{50}$ scores.

1.11

Dilution	Dilution	No. pos	No. neg	Infected (A)	Uninfected (B)	A+B	% infected	Index	TCID50/25 µl	TCID50/ml
10^1	10^-1	3	0	14	0	14	100%	0.253	1 x 10^5.253	4 x 10^6.253 7162423.4
10^2	10^-2	3	0	11	0	11	100%			
10^3	10^-3	3	0	8	0	8	100%			
10^4	10^-4	3	0	5	0	5	100%			
10^5	10^-5	2	1	2	1	3	67%			
10^6	10^-6	0	3	0	4	4	0%			
10^7	10^-7	0	3	0	7	7	0%			
10^8	10^-8	0	3	0	10	10	0%			

1.12

Dilution	Dilution	No. pos	No. neg	Infected (A)	Uninfected (B)	A+B	% infected	Index	TCID50/25 µl	TCID50/ml
10^1	10^-1	3	0	13	0	13	100%	0.746	1 x 10^4.746	4 x 10^5.746 2228742.99
10^2	10^-2	3	0	10	0	10	100%			
10^3	10^-3	3	0	7	0	7	100%			
10^4	10^-4	3	0	4	0	4	100%			
10^5	10^-5	1	2	1	2	3	33%			
10^6	10^-6	0	3	0	5	5	0%			
10^7	10^-7	0	3	0	8	8	0%			
10^8	10^-8	0	3	0	11	11	0%			

1.13

Dilution	Dilution	No. pos	No. neg	Infected (A)	Uninfected (B)	A+B	% infected	Index	TCID50/25 µl	TCID50/ml
10^1	10^-1	3	0	14	0	14	100%	0.253	1 x 10^5.253	4 x 10^6.253 7162423.41
10^2	10^-2	3	0	11	0	11	100%			
10^3	10^-3	3	0	8	0	8	100%			
10^4	10^-4	3	0	5	0	5	100%			
10^5	10^-5	2	1	2	1	3	67%			
10^6	10^-6	0	3	0	4	4	0%			
10^7	10^-7	0	3	0	7	7	0%			
10^8	10^-8	0	3	0	10	10	0%			

1.14

Dilution	Dilution	No. pos	No. neg	Infected (A)	Uninfected (B)	A+B	% infected	Index	TCID50/25 µl	TCID50/ml
10^1	10^-1	3	0	13	0	13	100%	0.746	1 x 10^4.746	4 x 10^5.746 2228742.99
10^2	10^-2	3	0	10	0	10	100%			
10^3	10^-3	3	0	7	0	7	100%			
10^4	10^-4	3	0	4	0	4	100%			
10^5	10^-5	1	2	1	2	3	33%			
10^6	10^-6	0	3	0	5	5	0%			
10^7	10^-7	0	3	0	8	8	0%			
10^8	10^-8	0	3	0	11	11	0%			

1.15

Dilution	Dilution	No. pos	No. neg	Infected (A)	Uninfected (B)	A+B	% infected	Index	TCID50/25 µl	TCID50/ml
10^1	10^-1	3	0	15	0	15	100%	0.500	1 x 10^5.500	4 x 10^6.500 12649110.64
10^2	10^-2	3	0	12	0	12	100%			
10^3	10^-3	3	0	9	0	9	100%			
10^4	10^-4	3	0	6	0	6	100%			
10^5	10^-5	3	0	3	0	3	100%			
10^6	10^-6	0	3	0	3	3	0%			
10^7	10^-7	0	3	0	6	6	0%			
10^8	10^-8	0	3	0	9	9	0%			

2.26

Dilution	Dilution	No. pos	No. neg	Infected (A)	Uninfected (B)	A+B	% infected	Index	TCID50/25 µl	TCID50/ml
10^1	10^-1	3	0	13	0	13	100%	0.746	1 x 10^4.746	4 x 10^5.746<br>2228742.996
10^2	10^-2	3	0	10	0	10	100%			
10^3	10^-3	3	0	7	0	7	100%			
10^4	10^-4	3	0	4	0	4	100%			
10^5	10^-5	1	2	1	2	3	33%			
10^6	10^-6	0	3	0	5	5	0%			
10^7	10^-7	0	3	0	8	8	0%			
10^8	10^-8	0	3	0	11	11	0%			

2.27

Dilution	Dilution	No. pos	No. neg	Infected (A)	Uninfected (B)	A+B	% infected	Index	TCID50/25 µl	TCID50/ml
10^1	10^-1	3	0	13	0	13	100%	0.746	1 x 10^4.746	4 x 10^5.746<br>2228742.996
10^2	10^-2	3	0	10	0	10	100%			
10^3	10^-3	3	0	7	0	7	100%			
10^4	10^-4	3	0	4	0	4	100%			
10^5	10^-5	1	2	1	2	3	33%			
10^6	10^-6	0	3	0	5	5	0%			
10^7	10^-7	0	3	0	8	8	0%			
10^8	10^-8	0	3	0	11	11	0%			

2.28

Dilution	Dilution	No. pos	No. neg	Infected (A)	Uninfected (B)	A+B	% infected	Index	TCID50/25 µl	TCID50/ml
10^1	10^-1	3	0	12	0	12	100%	0.500	1 x 10^4.500	4 x10^5.500<br>1264911.06
10^2	10^-2	3	0	9	0	9	100%			
10^3	10^-3	3	0	6	0	6	100%			
10^4	10^-4	3	0	3	0	3	100%			
10^5	10^-5	0	3	0	3	3	0%			
10^6	10^-6	0	3	0	6	6	0%			
10^7	10^-7	0	3	0	9	9	0%			
10^8	10^-8	0	3	0	12	12	0%			

2.29

Dilution	Dilution	No. pos	No. neg	Infected (A)	Uninfected (B)	A+B	% infected	Index	TCID50/25 µl	TCID50/ml
10^1	10^-1	3	0	16	0	16	100%	0.000	1 x 10^6.00	4 x 10^7<br>40000000.00
10^2	10^-2	3	0	13	0	13	100%			
10^3	10^-3	3	0	10	0	10	100%			
10^4	10^-4	3	0	7	0	7	100%			
10^5	10^-5	2	1	4	1	5	80%			
10^6	10^-6	2	1	2	2	4	50%			
10^7	10^-7	0	3	0	5	5	0%			
10^8	10^-8	0	3	0	8	8	0%			

2.30

Dilution	Dilution	No. pos	No. neg	Infected (A)	Uninfected (B)	A+B	% infected	Index	TCID50/25 µl	TCID50/ml
10^1	10^-1	3	0	12	0	12	100%	0.500	1 x 10^4.500	4 x 10^5.500<br>1264911.06
10^2	10^-2	3	0	9	0	9	100%			
10^3	10^-3	3	0	6	0	6	100%			
10^4	10^-4	3	0	3	0	3	100%			
10^5	10^-5	0	3	0	3	3	0%			
10^6	10^-6	0	3	0	6	6	0%			
10^7	10^-7	0	3	0	9	9	0%			
10^8	10^-8	0	3	0	12	12	0%			

3.41

Dilution	Dilution	No. pos	No. neg	Infected (A)	Uninfected (B)	A+B	% infected	Index	TCID50/25 µl	TCID50/ml
10^1	10^-1	3	0	12	0	12	100%			
10^2	10^-2	3	0	9	0	9	100%			
10^3	10^-3	3	0	6	0	6	100%			
10^4	10^-4	3	0	3	0	3	100%	0.500	1 x 10^4.500	
10^5	10^-5	0	3	0	3	3	0%			4 x 10^5.500
10^6	10^-6	0	3	0	6	6	0%			1264911.06
10^7	10^-7	0	3	0	9	9	0%			
10^8	10^-8	0	3	0	12	12	0%			

3.42

Dilution	Dilution	No. pos	No. neg	Infected (A)	Uninfected (B)	A+B	% infected	Index	TCID50/25 µl	TCID50/ml
10^1	10^-1	3	0	14	0	14	100%			
10^2	10^-2	3	0	11	0	11	100%			
10^3	10^-3	3	0	8	0	8	100%			
10^4	10^-4	3	0	5	0	5	100%	0.253	1 x 10^5.253	
10^5	10^-5	2	1	2	1	3	67%			4 x 10^6.253
10^6	10^-6	0	3	0	4	4	0%			7162423.41
10^7	10^-7	0	3	0	7	7	0%			
10^8	10^-8	0	3	0	10	10	0%			

3.43

Dilution	Dilution	No. pos	No. neg	Infected (A)	Uninfected (B)	A+B	% infected	Index	TCID50/25 µl	TCID50/ml
10^1	10^-1	3	0	13	0	13	100%			
10^2	10^-2	3	0	10	0	10	100%			
10^3	10^-3	3	0	7	0	7	100%			
10^4	10^-4	3	0	4	0	4	100%	0.746	1 x 10^4.746	
10^5	10^-5	1	2	1	2	3	33%			4 x 10^5.746
10^6	10^-6	0	3	0	5	5	0%			2228742.99
10^7	10^-7	0	3	0	8	8	0%			
10^8	10^-8	0	3	0	11	11	0%			

3.44

Dilution	Dilution	No. pos	No. neg	Infected (A)	Uninfected (B)	A+B	% infected	Index	TCID50/25 µl	TCID50/ml
10^1	10^-1	3	0	13	0	13	100%			
10^2	10^-2	3	0	10	0	10	100%			
10^3	10^-3	3	0	7	0	7	100%			
10^4	10^-4	3	0	4	0	4	100%	0.746	1 x 10^4.746	
10^5	10^-5	1	2	1	2	3	33%			4 x 10^5.746
10^6	10^-6	0	3	0	5	5	0%			2228742.996
10^7	10^-7	0	3	0	8	8	0%			
10^8	10^-8	0	3	0	11	11	0%			

3.45

Dilution	Dilution	No. pos	No. neg	Infected (A)	Uninfected (B)	A+B	% infected	Index	TCID50/25 µl	TCID50/ml
10^1	10^-1	3	0	15	0	15	100%			
10^2	10^-2	3	0	12	0	12	100%			
10^3	10^-3	3	0	9	0	9	100%			
10^4	10^-4	3	0	6	0	6	100%	0.500	1 x 10^5.500	
10^5	10^-5	2	1	3	1	4	75%			4 x 10^6.500
10^6	10^-6	1	2	1	3	4	25%			12649110.64
10^7	10^-7	0	3	0	6	6	0%			
10^8	10^-8	0	3	0	9	9	0%			

Table S8 MTT optical density in prevention model

1.11

Dilution	Raw OD			Mean	SEM
	1	2	3		
0.1	0.456	0.431	0.430	0.439	0.009
0.01	0.406	0.441	0.488	0.445	0.024
0.001	0.402	0.489	0.568	0.486	0.048
0.0001	0.812	0.810	0.906	0.843	0.032
0.00001	1.688	1.001	1.097	1.262	0.215
0.000001	1.470	1.555	1.527	1.517	0.025
0.0000001	1.782	1.633	1.597	1.671	0.057
0.00000001	1.943	1.629	1.546	1.706	0.121

1.12

Dilution	Raw OD			Mean	SEM
	1	2	3		
0.1	0.225	0.276	0.277	0.259	0.017
0.01	0.397	0.407	0.397	0.400	0.003
0.001	0.467	0.449	0.437	0.451	0.009
0.0001	0.742	0.633	0.690	0.688	0.031
0.00001	1.523	1.144	1.150	1.272	0.125
0.000001	1.564	1.262	1.305	1.377	0.094
0.0000001	1.636	1.670	1.522	1.609	0.045
0.00000001	1.760	1.676	1.635	1.690	0.037

1.13

Dilution	Raw OD			Mean	SEM
	1	2	3		
0.1	0.207	0.237	0.318	0.254	0.033
0.01	0.386	0.411	0.432	0.410	0.013
0.001	0.415	0.501	0.397	0.438	0.032
0.0001	0.852	0.962	0.770	0.861	0.056
0.00001	1.500	1.110	1.491	1.367	0.129
0.000001	1.538	1.664	1.446	1.549	0.063
0.0000001	1.633	1.585	1.558	1.592	0.022
0.00000001	1.808	1.656	1.361	1.608	0.131

1.14

Dilution	Raw OD			Mean	SEM
	1	2	3		
0.1	0.158	0.167	0.158	0.161	0.003
0.01	0.347	0.471	0.457	0.425	0.039
0.001	0.621	0.513	0.476	0.537	0.043
0.0001	0.728	0.638	0.726	0.697	0.030
0.00001	1.452	1.154	1.163	1.256	0.098
0.000001	1.143	1.815	1.372	1.443	0.197
0.0000001	1.587	1.902	1.171	1.553	0.212
0.00000001	1.651	1.866	1.517	1.678	0.102

1.15

Dilution	Raw OD			Mean	SEM
	1	2	3		
0.1	0.112	0.124	0.112	0.116	0.004
0.01	0.405	0.155	0.348	0.303	0.076
0.001	0.400	0.508	0.347	0.418	0.047
0.0001	0.549	0.927	0.537	0.671	0.128
0.00001	1.472	1.098	1.060	1.210	0.131
0.000001	1.506	1.574	1.628	1.569	0.035
0.0000001	1.633	1.851	1.600	1.695	0.079
0.00000001	1.718	1.757	1.724	1.733	0.012

2.26

Dilution	Raw OD			Mean	SEM
	1	2	3		
0.1	0.211	0.229	0.230	0.223	0.006
0.01	0.441	0.582	0.362	0.462	0.064
0.001	0.478	0.552	0.509	0.513	0.021
0.0001	0.604	0.988	0.612	0.735	0.127
0.00001	1.581	1.146	0.926	1.218	0.192
0.000001	1.662	1.600	1.035	1.432	0.199
0.0000001	1.512	1.417	1.540	1.490	0.037
0.00000001	1.694	1.779	1.711	1.728	0.026

2.27

Dilution	Raw OD			Mean	SEM
	1	2	3		
0.1	0.183	0.192	0.238	0.204	0.017
0.01	0.514	0.556	0.524	0.531	0.013
0.001	0.550	0.441	0.595	0.529	0.046
0.0001	0.646	0.569	0.979	0.731	0.126
0.00001	1.166	1.167	1.645	1.326	0.160
0.000001	1.546	1.257	1.477	1.427	0.087
0.0000001	1.566	1.813	1.772	1.717	0.076
0.00000001	1.665	1.797	1.976	1.813	0.090

2.28

Dilution	Raw OD			Mean	SEM
	1	2	3		
0.1	0.250	0.267	0.330	0.282	0.024
0.01	0.456	0.379	0.497	0.444	0.035
0.001	0.532	0.365	0.490	0.462	0.050
0.0001	0.730	0.844	0.681	0.752	0.048
0.00001	0.911	1.306	1.480	1.232	0.168
0.000001	1.633	1.449	1.394	1.492	0.072
0.0000001	1.605	1.640	1.774	1.673	0.052
0.00000001	1.597	1.615	1.883	1.698	0.092

2.29

Dilution	Raw OD			Mean	SEM
	1	2	3		
0.1	0.193	0.220	0.252	0.222	0.017
0.01	0.400	0.353	0.534	0.429	0.054
0.001	0.515	0.439	0.512	0.489	0.025
0.0001	0.725	0.562	0.759	0.682	0.061
0.00001	1.088	0.631	1.249	0.989	0.185
0.000001	1.145	1.484	1.177	1.269	0.108
0.0000001	1.594	1.623	1.059	1.425	0.183
0.00000001	1.698	1.685	1.690	1.691	0.004

2.30

Dilution	Raw OD			Mean	SEM
	1	2	3		
0.1	0.191	0.209	0.264	0.221	0.022
0.01	0.460	0.497	0.529	0.495	0.020
0.001	0.485	0.490	0.559	0.511	0.024
0.0001	0.819	0.898	1.151	0.956	0.100
0.00001	1.684	1.144	1.351	1.393	0.157
0.000001	1.669	1.573	1.623	1.622	0.028
0.0000001	1.691	1.313	1.845	1.616	0.158
0.00000001	1.755	1.739	1.373	1.622	0.125

3.41

Dilution	Raw OD			Mean	SEM
	1	2	3		
0.1	0.180	0.300	0.278	0.253	0.037
0.01	0.412	0.477	0.455	0.448	0.019
0.001	0.427	0.443	0.478	0.449	0.015
0.0001	0.743	1.122	0.881	0.915	0.111
0.00001	1.250	1.526	1.789	1.522	0.156
0.000001	1.643	1.721	1.284	1.549	0.135
0.0000001	1.748	1.800	1.780	1.776	0.015
0.00000001	1.674	1.720	1.300	1.565	0.133

3.42

Dilution	Raw OD			Mean	SEM
	1	2	3		
0.1	0.151	0.144	0.174	0.156	0.009
0.01	0.451	0.087	0.481	0.340	0.127
0.001	0.495	0.432	0.537	0.488	0.031
0.0001	0.568	0.596	0.650	0.605	0.024
0.00001	0.708	0.754	0.996	0.819	0.089
0.000001	1.577	1.685	1.295	1.519	0.116
0.0000001	1.756	1.269	1.020	1.348	0.216
0.00000001	1.257	1.717	1.716	1.563	0.153

3.43

Dilution	Raw OD			Mean	SEM
	1	2	3		
0.1	0.291	0.258	0.292	0.280	0.011
0.01	0.444	0.475	0.517	0.479	0.021
0.001	0.492	0.505	0.533	0.510	0.012
0.0001	0.875	0.622	0.822	0.773	0.077
0.00001	1.410	1.643	1.186	1.413	0.132
0.000001	1.479	1.343	1.387	1.403	0.040
0.0000001	1.330	1.269	1.468	1.356	0.059
0.00000001	1.624	1.801	1.074	1.500	0.219

3.44

Dilution	Raw OD			Mean	SEM
	1	2	3		
0.1	0.199	0.198	0.193	0.197	0.002
0.01	0.176	0.170	0.535	0.294	0.121
0.001	0.515	0.512	0.582	0.536	0.023
0.0001	0.739	0.559	0.723	0.674	0.058
0.00001	1.360	0.792	1.367	1.173	0.191
0.000001	1.115	1.564	1.110	1.263	0.151
0.0000001	1.560	1.776	1.148	1.495	0.184
0.00000001	1.597	1.618	1.403	1.539	0.068

3.45

Dilution	Raw OD			Mean	SEM
	1	2	3		
0.1	0.167	0.202	0.193	0.187	0.010
0.01	0.428	0.344	0.446	0.406	0.031
0.001	0.445	0.424	0.584	0.484	0.050
0.0001	0.936	0.636	0.788	0.787	0.087
0.00001	1.046	1.348	1.339	1.244	0.099
0.000001	1.453	1.188	1.163	1.268	0.093
0.0000001	1.677	1.560	1.218	1.485	0.138
0.00000001	1.708	1.716	1.341	1.588	0.124

Figure S1. Representative images of lung histopathology scores and area affected at termination in treatment model.

Mouse 1.3 (x2.5 and x20). PR8 infection, control.
Severe bronchiolitis with lumen filled with inflammatory cells and debris (1), haemorrhage (2), interstitial and alveolar inflammation(3)

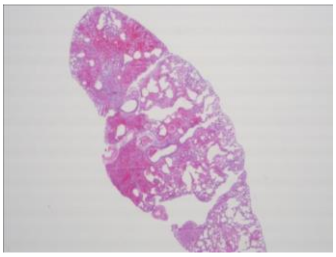

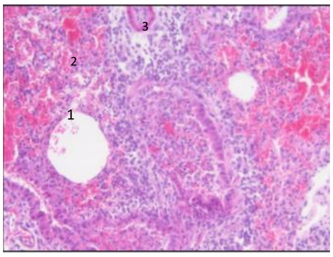

Mouse 2.11 (x2.5 and x20). PR8 infection, fucoidan at 3.52mg/day.

Focal bronchiolar epithelial disruption and inflammation(1); consolidation of alveolar tissue (2).

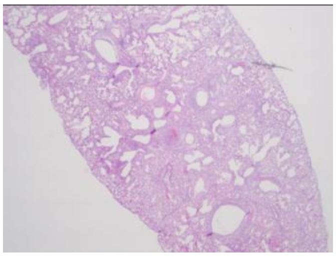

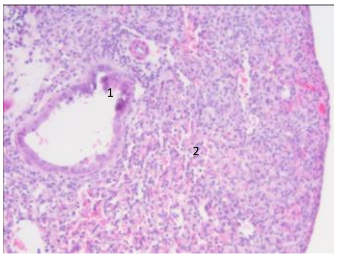

Figure S2. Mean $TCID_{50}$ values of lung homogenates. Data are presented as mean $TCID_{50}$ of group (n = 5) ± SEM.

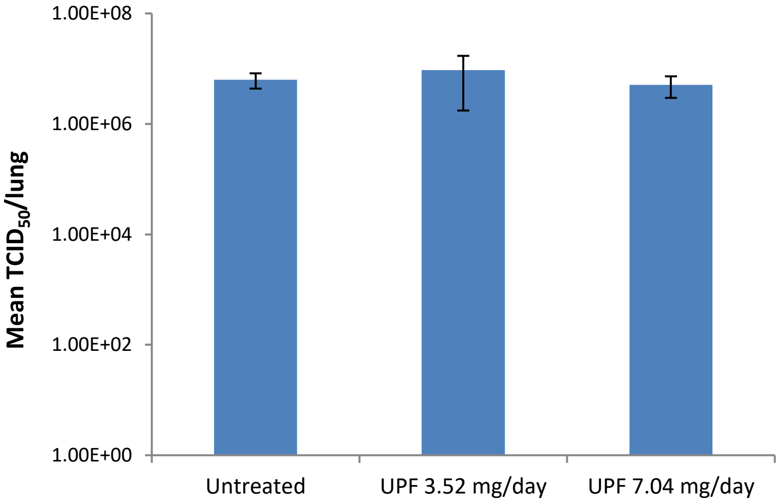

Supplement: Supplementary file 1 [file marinedrugs-18-00246-s001.pdf]
